# Nitro- and nitrooxy-organic inhibitors of methanogenesis: revealing knowledge gaps and alternate ways in bovine rumen microbiome metabolism

**DOI:** 10.1128/aem.02442-25

**Published:** 2026-08-04

**Authors:** Biswarup Mukhopadhyay

**Affiliations:** 1 Department of Biochemistry, Virginia Tech 1757, Blacksburg, Virginia, USA; 2 Genetics, Bioinformatics, and Computational Biology Ph.D. Program, Virginia Tech 1757, Blacksburg, Virginia, USA; 3 Virginia Tech Carilion School of Medicine, Virginia Tech 1757, Blacksburg, Virginia, USA; University of Nebraska-Lincoln, Lincoln, Nebraska, USA

**Keywords:** rumen, bovine, microbiome, methane, methanogen, methanogenesis, inhibitor, hydrogen, nitroorganic, nitrooxyorganic, electron sink, electron transfer, anaerobic microbiology, metabolism

## Abstract

In the rumen, methanogens consume H_2_, generating methane and thermodynamically facilitating the production of short-chain fatty acids (SCFAs), ruminants’ main energy source. Yet in animal trials, inhibiting methanogenesis by 27%–90% with 3-nitrooxypropanol minimally perturbs ruminal SCFA levels, sparing disproportionately low amount of H_2_. An *Applied and Environmental Microbiology* article (A. Castaneda, N. Indugu, K. Challa, K. Narayan, et al., Appl Environ Microbiol e01033-25, 2025, https://journals.asm.org/doi/10.1128/aem.01033-25) reports similar outcomes in an *in vitro* rumen experiment where ethyl-nitroacetate and ethyl-2-nitropropionate inhibited methanogenesis 100%. Hence, the rumen has fallback ways, perhaps evolved through exposures to plant metabolites. The nitroorganics offer an opportunity to determine the consequences of 100% inhibition of ruminal methanogenesis in live animals.

## COMMENTARY

The ruminant animals, including livestock such as cows, sheep, and goats that constitute major components of the agriculture industry, derive nutrition from microbial digestion of feed in their foregut called the rumen (1). Here, a group of bacteria and anaerobic fungi and protozoa oxidize carbohydrates, the major component of ruminant’s feed (2), often producing pyruvate and reducing equivalents or [H] units and ATP (1, 3–5) (reaction 1, from glucose); [H] is equivalent to one electron (e^−^) and one proton (H^+^) combined or ½H_2_. Some of the pyruvate and [H] pool is utilized to produce three short-chain fatty acids or SCFAs (acetate, propionate, and butyrate) that provide as high as 80% of the energy nutrition for the animals (6). Oxidation of pyruvate leads to an equimolar amount of acetyl-CoA and formate or CO_2_ + 2[H] (reactions 2 and 3) (5), and acetyl-CoA serves as a precursor for acetate (reaction 4) and butyrate (reaction 5); the overall pyruvate to butyrate conversion is redox neutral (reaction 5). In the reactions presented below, P_i_ (inorganic phosphate) has been considered equivalent to HPO_4_^2−^ and the terminal phosphate group of a nucleotide as fully ionized; these considerations refer to physiologically relevant pH values of 7.4–7.8 (7).


(1) $$C_{6}H_{12}O_{6}+2ADP+2P_{i}\to 2{CH}_{3}-CO-{COO}^{-}+4[H]+2ATP+2H_{2}O$$

(2) $${CH}_{3}\text{-}CO\text{-}{COO}^{-}+H^{+}+HS\text{-}CoA\to {CH}_{3}\text{-}CO\text{-}SCoA+{CO}_{2}+2[H]$$

(3) $${CH}_{3}\text{-}CO\text{-}{COO}^{-}+H^{+}+HS\text{-}CoA\to {CH}_{3}\text{-}CO\text{-}SCoA+{HCOO}^{-}+H^{+}$$

(4) $${CH}_{3}\text{-}CO\text{-}SCoA+ADP+P_{i}\to {CH}_{3}\text{-}{COO}^{-}+HS\text{-}CoA+ATP$$

(5) $$2{CH}_{3}\text{-}CO\text{-}{COO}^{-}+2H^{+}+ADP+P_{i}\to {CH}_{3}\text{-}{CH}_{2}\text{-}{CH}_{2}\text{-}{COO}^{-}+2{CO}_{2}+ATP+H_{2}O$$

Propionate is generated primarily via the succinate-propionate pathway, initiating with phosphoenolpyruvate via either phosphoenolpyruvate carboxylase (reaction 6) or phosphoenolpyruvate carboxykinase ( reactions 7A and 7B, GDP- and ADP-dependent) and with pyruvate via pyruvate carboxylase (reaction 8), and consuming 4[H] per mol of product formed (1, 4, 5). The same pathway also operates with lactate as the starting point (reaction 9). The second-ranking propionate production system, acrylate pathway, involves two organisms wherein lactate produced from pyruvate by the first organism (reaction 10) is processed by the second (reaction 11) (5). The propanediol pathway, converting lactaldehyde, derived from lactate or sugar degradation (8), constitutes a minor route of propionate synthesis in the rumen (4); it provides a way to utilize deoxy-sugars (8).


(6) $${CH}_{2}\text{=}C({OPO}_{3}{}^{2-})\text{-}{COO}^{-}+ADP+4[H]+H^{+}\to {CH}_{3}\text{-}{CH}_{2}\text{-}{COO}^{-}+ATP+H_{2}O$$

(7A) $${CH}_{2}\text{=}C({OPO}_{3}{}^{2-})\text{-}{COO}^{-}+2H^{+}+4[H]+GDP+ADP+P_{i}\to {CH}_{3}\text{-}{CH}_{2}\text{-}{COO}^{-}+GTP+ATP+2H_{2}O$$

(7B) $${CH}_{2}\text{=}C({OPO}_{3}{}^{2-})\text{-}{COO}^{-}+2H^{+}+4[H]+2ADP+P_{i}\to {CH}_{3}\text{-}{CH}_{2}\text{-}{COO}^{-}+2ATP+H_{2}O$$

(8) $${CH}_{3}\text{-}CO\text{-}{COO}^{-}+4[H]\to {CH}_{3}\text{-}{CH}_{2}\text{-}{COO}^{-}+H_{2}O$$

(9) $${CH}_{3}\text{-}CH(OH)\text{-}{COO}^{-}+2[H]+H^{+}+ADP+P_{i}\to {CH}_{3}\text{-}{CH}_{2}\text{-}{COO}^{-}+ATP+2H_{2}O$$

(10) $${CH}_{3}\text{-}CO\text{-}{COO}^{-}+2[H]\to {CH}_{3}\text{-}CH(OH)\text{-}{COO}^{-}$$

(11) $$3{CH}_{3}\text{-}CH(OH)\text{-}{COO}^{-}\to 2{CH}_{3}\text{-}{CH}_{2}\text{-}{COO}^{-}+{CH}_{3}\text{-}{COO}^{-}+{CO}_{2}+H_{2}O$$

The leftover or excess [H], which is mostly released as hydrogen gas (H_2_), thermodynamically blocks the very feed degradation process that produces it, and methanogenic archaea or methanogens consume this product, producing methane (reaction 12) and thereby removing the block (1, 3, 4). Under certain nutritional conditions, [H] is directed toward the formation of formate via the action of formate dehydrogenase which is then converted to methane by methanogens (reaction 13) (9, 10); the importance of this route of [H] disposal in the rumen is still not clear (10). Under high H_2_ partial pressure, some of the acetyl-CoA is converted to ethanol, consuming 4[H] equivalent/mol (reaction 14). The above- described overall fine-tuned interspecies electron or reductant transfer-based system (1, 3, 4, 10) is a result of 50 million years of host-microbiome coevolution (11). It provides the ruminants with their nutrients (1), and the animals belch out methane (1, 4).


(12) $$4H_{2}+{CO}_{2}\to {CH}_{4}+2H_{2}O$$

(13) $$4{HCOO}^{-}+4H^{+}\to {CH}_{4}+3{CO}_{2}+2H_{2}O$$

(14) $${CH}_{3}\text{-}CO\text{-}SCoA+4[H]\to {CH}_{3}\text{-}{CH}_{2}\text{-}OH+HS\text{-}CoA$$

(15) $$\begin{array}{rl} & {CH}_{3}\text{-}X+H_{2}\to {CH}_{4}+HX\\ & \left(X=\text{-}OH,\text{-}{NH}_{2},\text{ and }\text{-}SH\right) \end{array}$$

**Fig 1:**
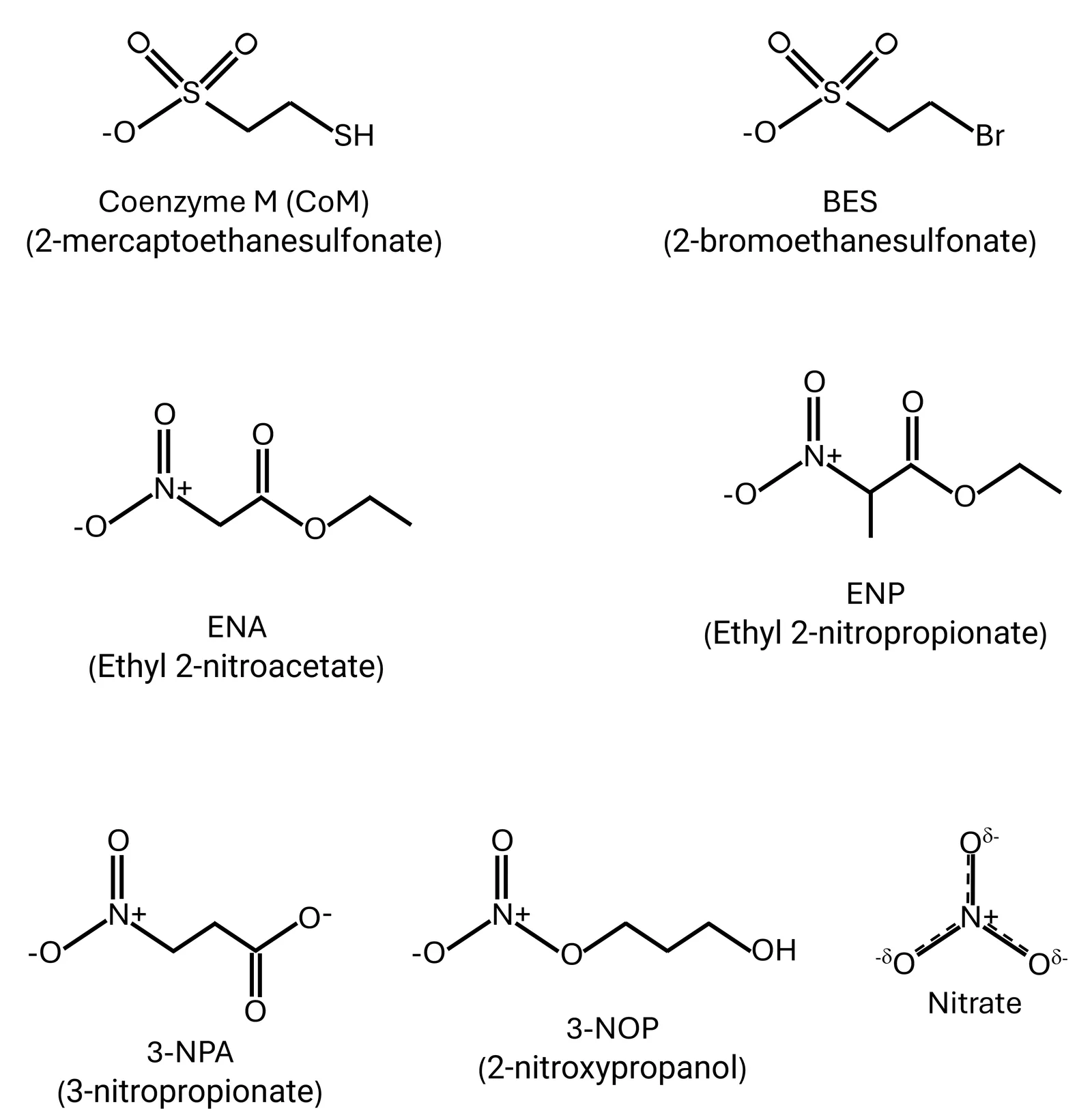
Structures of coenzyme M and select inhibitors of methanogenesis. BES is the most studied inhibitor of methanogenesis (4, 17).

Methane is a potent greenhouse gas and a high-energy molecule, and therefore, reducing its emission from the livestock would help to slow down global warming and to lessen energy loss, thereby, improving feed utilization efficiency for the animals (12, 13). For this rationale, there have been efforts to block the methanogenesis branch of this system (14). The commonly investigated avenues are providing an alternate electron or [H] sink such as nitrate, sulfate, and fumarate (Avenue 1) and inhibiting methanogens with chemicals provided either directly or as contained in microbial or plant biomass (Avenue 2) (14). This commentary looks at the learning from studies by Castaneda et al. (15), where the utility of two nitroorganics, ethyl-nitroacetate (ENA) and ethyl-2-nitropropionate (ENP) (Fig. 1), in inhibiting methane formation by rumen microbiome has been examined *in vitro*. It also integrates knowledge gathered from an earlier study with ENA (16) and animal-based investigations with 3-nitrooxypropanol (3-NOP) (Fig. 1), a nitroxy-derivative that is the best-performing rumen methanogenesis inhibitor in the field and has been commercialized as Bovaer by DSM-Firmenich AG (Kaiseraugst, Switzerland) (17–23). The target of 3-NOP, an analog of coenzyme M (Fig. 1), is methyl-coenzyme M reductase, which is required for methanogenesis from all methanogenic substrates (4, 17). Since methanogenesis is the sole source of energy for methanogens, methyl-coenzyme M reductase is essential to these organisms (4). It seems that ENA and ENP target both the methanogens and fermenting bacteria (15, 16) and, therefore, provide potentially an Avenue 3 for blocking methanogenesis in rumen.

Castaneda et al. (15) found that in an *in vitro* rumen system composed of buffer-supplemented rumen fluid containing feed particles, ENA and ENP caused complete (100%) inhibition of methane production; this activity of ENA was reported previously (16). With nitrate (Fig. 1), a positive control, the extent of inhibition, was 50% (15). However, the two nitroorganics impacted other aspects of rumen metabolism differently. The addition of ENA caused significant accumulation of hydrogen, but accounting for only 1/4th of what would be expected from the observed inhibition of methanogenesis (15). A minor rise in propionate and butyrate levels (10% and 14%, respectively), even lesser impacts on longer and branched-chain fatty acids, and a significant drop in the total VFA level suggest that the unaccounted [H] was not directed toward the synthesis of VFAs. With ENP, a minor hydrogen buildup and little changes in the propionate and butyrate levels (−4.2% and 0.8%, respectively) (15) presented a more complicated case. In both cases, acetate level dropped (24% with ENA and 7% with ENP), perhaps due to hydrogen accumulation (reaction 14) (15), indirectly indicating that ENP did cause some accumulation of hydrogen as well. The lack of significant hydrogen accumulation and changes in the fatty acid profile with nitrate provided a validation for the experimental system (15), as this oxyanion provides an alternate [H] disposal pathway that produces nitrite and ammonia (15).

The three observations that ENA significantly affected the abundance of *Methanobrevibacter ruminantium* M1, a predominant rumen methanogen (24), allowed *Methanosphaera stadtmanae* to proliferate better upon a longer exposure, and did not impact strain ISO4-H5, a bovine rumen isolate belonging to the order *Methanomassiliicoccales* (25), provide a clue to the mode of action of this nitroorganic on methanogens. *M. ruminantium* is a CO_2_-reducing hydrogenotroph that grows on both H_2_ + CO_2_ and formate (26) (reactions 12 and 13) and *M. stadtmanae* and ISO4-H5 rely on methyl-reducing hydrogenotrophic methanogenesis (reaction 15; X being -OH for both organisms and -NH_2_ for ISO4-H5) (4, 25, 27). It is likely that ENA affected the growth of *M. ruminantium* by inhibiting hydrogen utilization enzymes, and in *M. stadtmanae* and ISO4-H5, such units are either resistant to ENA or a lowered hydrogen utilization rate does not appreciably limit their energy production activities. Indeed, in terms of hydrogen utilization, the methyl-reducing and CO_2_-reducing hydrogenotrophs are distinct (4). A previous study found that ENA improves the fermentation efficiency in an *in vitro* rumen system a bit and suggested that it inhibits the hydrogen uptake activity of the methanogens and not the hydrogen production by the fermenters (16). With ENP, there are possibilities of direct inhibitory actions on both methanogens and fermenting microorganisms as well as feedback inhibition on hydrogen production. It remains to be investigated if ENA and ENP could serve as alternate electron acceptors. This possibility has not seen with a related compound, 3-nitropropionate (3-NPA) (Fig. 1), as the inhibition of methane production did not increase with the amount of the compound added (28). Some uncertainties about the modes of action of these nitroorganics also come from the variations in the outcomes of *in vitro* studies. In their initial experiments, Castaneda et al. (15) found that *Methanosphaera* species present in the rumen sample were inhibited by ENA and ENP, and then in the final tests, the respective abundances increased by an exposure to ENA. The pure culture isolate of *M. stadtmanae* was equally sensitive to both nitroorganics in terms of growth and methane production (15). This difference between the impacts of ENA and ENP on *M. stadtmanae*, a pure culture isolate from human feces (27), and *Methanosphaera* species in the rumen microbiome suggests that these compounds act differently on *Methanosphaera* species from different sources. The effect could also arise because these compounds act on both the methanogens and non-methanogen members of the microbiome, generating a complex outcome (15). Indeed, in the case investigated by Castaneda et al., these nitroorganics restructured the composition of the bacterial segment of the microbiome (15).

The results of animal-based studies with 3-NOP also highlight serious gaps in our knowledge of rumen microbiome metabolism (18–23). This nitrooxyorganic compound provides up to 27%–90% reduction in methane emission from beef and dairy cattle, and here also, the respective unutilized [H] is not fully accounted for by the free H_2_ that is belched out (18–21, 23, 29). Ideally, for the elimination of one mol of CH_4_ production through methanogenesis from H_2_ + CO_2_, formate, or H_2_ + methanol, 4, 4, or 1 mol of H_2_ equivalent would be left behind, respectively; this calculation assumes that in the absence of its use, each mole of formate is oxidized to an equimolar amount of H_2_. However, the amount of H_2_ emitted by dairy cows fed with 3-NOP is only a very small fraction of that is expected, and it goes down further as the treatment progresses (18, 22). A calculation with the data reported from a recent study with dairy calves, where a higher amount of spared H_2_ was detected, showed that the ratio between the increase in H_2_ emission and decrease in CH_4_ emission to be in the 1–1.3 range (20). It means that 68%–75% of the [H] pool that was not used for methanogenesis was directed toward another electron sink. This calculation considers that most of the methanogens of this system were CO_2_-reducing hydrogenotrophs (20) that consume 4 mol of H_2_ to generate 1 mol of CH_4_. The value of unaccounted [H] would be lower than 68%–75% if the methyl-reducing hydrogenotrophic members were significant contributors to methanogenesis. The observed impacts of 3-NOP on the microbes suggest the former, a higher value for the diverted [H]. The pure culture isolates of the methyl-reducing hydrogenotrophic methanogens are generally 4- to 40-fold less sensitive to 3-NOP than the CO_2_-reducing hydrogenotrophic members of the group (17, 20). Also, both the metagenomic and metatranscriptomic data show that 3-NOP affects CO_2_-reducing hydrogenotrophic population in the rumen substantially, whereas a weaker effect is seen for methyl-reducing hydrogenotrophs (20, 22). The residual methane production in 3-NOP treated rumen can be attributed to the latter group (20).

The bacterial community response to 3-NOP also presents a mixed picture. A concerted action is seen to lower the use of H_2_ production routes, such as A3 [FeFe]-hydrogenases, that require special efforts involving electron bifurcation or confurcation, and to deploy those with the ability to function under high H_2_ levels, such as monomeric ferredoxin-dependent group A1 and B [FeFe]-hydrogenases (20, 22). Similarly, certain highly abundant fermentative organisms elevate the expression of genes linked to butyrate, propionate, ethanol, and lactate production or even for directing pyruvate toward formate generation via pyruvate formate lyase (reaction 3), and thereby, avoiding H_2_ accumulation through pyruvate-ferredoxin oxidoreductase-hydrogenase combination (reaction 2) (20). Then, one sees two contrasting actions for acetate production (20): transcript levels being lowered for the fermentative route and elevated for the reductive path; an increase in the abundances of putative reductive acetogen species from *Oscillospiraceae, Lachnospiraceae*, and *Christensenellaceae* families that could help to sequester H_2_. In another study, 3-NOP administration initially lowered the abundance of *Lachnospiraceae* and then raised it with time and impacted many other potentially SCFA-producing bacteria either similarly or in an opposite manner (22). Despite all these actions, an excess [H] driven significant rise in the SCFA levels was not seen (20, 22), except in one case where the butyrate level rose by 18%–26% even though the total VFA level decreased by 6%–15% (22). A drop in the level of acetate (20, 22) suggested that an enhanced reductive acetogenesis activity could not make up for the drop in the heterotrophic route’s throughput.

The outcomes from work with nitro- and nitrooxy-organic inhibitors highlight the incompleteness of our knowledge about the metabolism of seemly stable rumen microbiome. While a significant advancement toward filling this gap would require longer-term research, in the shorter-term mechanistic studies with ENA and ENP (15, 16) could provide a clearer view of the Avenue 3 as mentioned above. The possibilities along these lines and others are discussed below.

The differences in the sensitivities of *M. ruminantium* M1, *M. stadtmanae*, and ISO4-H5 to ENA and ENP (15) provide an effective comparative basis for both focused physiological studies and omics-based broad investigations for identifying and characterizing the ENA and ENP targets. These efforts could be followed up with molecular level interrogations on inhibitor-target interactions, as have been done with 3-NOP (17). The data from Castaneda et al. (15) also highlight the need for precision *in vitro* pure culture studies. Their experiments with *M. stadtmanae* cultures involved the use of Hungate tubes with a mixture of H_2_ and CO_2_ (80:20, volume/volume; 1 bar) and an aliquot of methanol as substrates. Considering that *Methanosphaera* cannot oxidize methanol to CO_2_ and methanogenesis is its only source of energy (27), the cultures with ENA and ENP would be in stasis. Indeed, the cultures did not produce CH_4_. However, the volumes of H_2_ and CO_2_ present in the tubes varied and the respective ratios fell in the range of 0.75:1 to 1.9:1 instead of remaining constant at 4:1 (Table 6 of reference 15). An explanation for this observation is that the tubes developed leaks, or the inhibitors instigated a new type of metabolism in the organism.

A full accounting of how the excess [H] originating from the inhibition of methanogenesis is utilized would require detailed investigations on both the microbiome and host sides, where lie many unclear areas. For example, if the rates of absorption of SCFAs from the rumen are much higher than that of their production, then the observed levels of these compounds in an uninhibited rumen microbiome represent the respective thresholds for absorption. Under this situation, the use of excess [H] for the synthesis of SCFAs may not easily alter their steady-state levels in the rumen. This scenario will make the enhancement in SCFA production as a trusted standby thermodynamic block remover for the fermentation that comes into operation only when the methanogens, the high-affinity H_2_ utilizers (9), are removed from action. A similar situation would be expected if a new or enhanced need for a particular use of acetate or acetyl-CoA comes up when methanogens are inhibited. It may even call for an elevated level of acetyl-CoA synthesis activity. These hypotheses are consistent with the findings that under 3-NOP challenge, the expression of genes linked to fermentative production of butyrate and propionate and reductive acetyl-CoA synthesis increase (20); for the latter, even new organisms are called into action (20). Here, the initiation or enhancement of the production of longer-chain VFAs such as valerate, isovalerate, and caproate could provide a helping hand; indeed, redox manipulation and inclusion of intermediates such as ethanol are known to stimulate the production of long-chain fatty acids by bovine rumen contents via reverse β-oxidation (30). However, the observed changes in the respective levels due to the inhibition of methanogenesis (15, 20) are too small for this route to be significant unless the argument of rapid uptake by the host is invoked. Here, it is noted that the information on the *in situ* SCFA production and absorption rates in the rumen has been hard to generate (31). Observing a rise in the butyrate level under 3-NOP challenge in an animal trial, it has been proposed that the production of this SCFA can act as a route for the disposal of spared H_2_ (22). This is an unlikely explanation as the overall process of the production of butyrate from glucose leads to excess [H], and this situation would be independent of the intermediate, such as ethanol, that the process runs through (5, 32).

The microbial biomass synthesis could serve as a significant [H] sink as well. According to Beauchemin and Thauer, this process seems to be insignificant as the redox status of the substrate (feed carbohydrates) and cell carbon is near zero (31). However, this route becomes reasonable if rumen microbes synthesize lipids, which are more reduced than carbohydrates, as storage compounds, and there are hints for this possibility. *Neocallimastix frontalis*, a bovine rumen anaerobic fungus, synthesize triacylglycerol and sterols (33). Certain anaerobic bacteria produce specialized lipids such as plasmalogens employing anaerobic environment-specific systems (34). A comprehensive analysis of the metatranscriptome and metabolite data for the microbiome under inhibitor challenge could help to determine if these lipid synthesis activities occur at significant levels. Also, such high-energy materials contained within microbial cells could escape the downstream processing and end up in the manure, and to the knowledge of this author, there has not been an effort to determine if the feeding of a methanogenesis inhibitor changes the energy content of the manure. A meta-analysis has shown that while feeding 3-NOP does not improve body weight much in dairy cows, it increases the fat and protein contents of milk (19).

There are several understudied parts of the rumen microbiome that could harbor non-methanogenic [H] sinks. Anaerobic fungi and protozoa which constitute up to 20% and 50% of the rumen microbial biomass, respectively, and impact feed digestibility, microbial protein supply, and methane production in the rumen (35, 36) represent such components. As mentioned above, their lipid synthesis activities could serve as hydrogen sinks (33). Their hydrogenosomes feed hydrogen to methanogens and bacteria (37), and they could be participating in cross-feeding activities such as those exemplified by the lactic acid bacteria*—Megasphaera elsdenii* collaboration, where the latter consumes lactic acid produced by the former (38), and the production of butyrate, caproate, and H_2_ by *Clostridium kluyveri* with ethanol and acetate supplied by other microbes (32). Even the rumen archaea and bacteria that respond to the exposure of the nitro- and nitrooxy-compounds in most parts remain to be characterized for their lower-level taxonomic identities, metabolic and physiological potentials, and *in situ* actions (15, 20). Here, efforts to isolate these organisms into pure cultures or even the development of respective low-complexity enrichments or mixed cultures and characterize their metabolic properties would bring major help toward achieving the goal of nutritional or inhibitor-based remodeling of rumen microbiome metabolism toward a desired end.

As a closing comment, it is noted that the work with the inhibitors teach us that rumen microbiome system leverages methanogenesis for a better efficiency, but it can function well enough without it. It is possible that in the wild the ruminants are exposed to methanogenesis inhibitors, such as bromoform and tannins, present in plant material that they feed on (14), and such exposures have helped them to develop alternate ways of operating the rumen system. Once the respective details are elucidated, these could be leveraged for bringing benefits for the animals and environment. The knowledge gained from partial shutdown of methanogenesis by a nitrooxyorganic now could be put to the test and expanded via animal studies with nitroorganics that cause total inhibition of methanogenesis in an *in vitro* rumen system (15); these studies will help to determine whether the bovine rumen system could be made fully free of methanogens.
